# Neurotrophic tyrosine receptor kinase gene fusions in adult and pediatric patients with solid tumors: a clinicogenomic biobank and record linkage study of expression frequency and patient characteristics from Finland

**DOI:** 10.2340/1651-226X.2024.26452

**Published:** 2024-07-05

**Authors:** Wei Zhang, Arndt A. Schmitz, Roosa E. Kallionpää, Merja Perälä, Niina Pitkänen, Mikko Tukiainen, Erika Alanne, Korinna Jöhrens, Renate Schulze-Rath, Bahman Farahmand, Jihong Zong

**Affiliations:** a Bayer US LLC, Whippany, NJ, USA; b Bayer AG, Berlin, Germany; c Auria Biobank, University of Turku and Turku University Hospital, Turku, Finland; d Department of Oncology and Radiotherapy, Turku University Hospital, Turku, Finland; e Western Finland Cancer Centre, Turku, Finland; f Dresden University Hospital, Technical University Dresden, Dresden, Germany; g Bayer AB, Solna, Sweden

**Keywords:** Clinicogenomic, *NTRK* gene fusions, solid tumors, pediatrics, epidemiology, biobank, Finland

## Abstract

**Background:**

Neurotrophic tyrosine receptor kinase (*NTRK*) gene fusions are oncogenic drivers. Using the Auria Biobank in Finland, we aimed to identify and characterize patients with these gene fusions, and describe their clinical and tumor characteristics, treatments received, and outcomes.

**Material and methods:**

We evaluated pediatrics with any solid tumor type and adults with colorectal cancer (CRC), non-small cell lung cancer (NSCLC), sarcoma, or salivary gland cancer. We determined tropomyosin receptor kinase (TRK) protein expression by pan-TRK immunohistochemistry (IHC) staining of tumor samples from the Auria Biobank, scored by a certified pathologist. *NTRK* gene fusion was confirmed by next generation sequencing (NGS). All 2,059 patients were followed-up starting 1 year before their cancer diagnosis.

**Results:**

Frequency of *NTRK* gene fusion tumors was 3.1% (4/127) in pediatrics, 0.7% (8/1,151) for CRC, 0.3% (1/288) for NSCLC, 0.9% (1/114) for salivary gland cancer, and 0% (0/379) for sarcoma. Among pediatrics there was one case each of fibrosarcoma (*TPM3::NTRK1)*, Ewing’s sarcoma (*LPPR1::NTRK2)*, primitive neuroectodermal tumor (*DAB2IP::NTRK2)*, and papillary thyroid carcinoma (*RAD51B::NTRK3*). Among CRC patients, six harbored tumors with *NTRK1* fusions (three fused with *TPM3*), one harbored a *NTRK3::GABRG1* fusion, and the other a *NTRK2::FXN/LPPR1* fusion. Microsatellite instability was higher in CRC patients with *NTRK* gene fusion tumors versus wild-type tumors (50.0% vs. 4.4%). Other detected fusions were *SGCZ::NTRK3* (NSCLC) and *ETV6::NTRK3* (salivary gland cancer). Four patients (three CRC, one NSCLC) received chemotherapy; one patient (with CRC) received radiotherapy.

**Conclusion:**

*NTRK* gene fusions are rare in adult CRC, NSCLC, salivary tumors, sarcoma, and pediatric solid tumors.

## Introduction

Fusions involving a gene of the neurotrophic tyrosine receptor kinase (*NTRK*) family are well-known oncogenic drivers of diverse cancers in adult and pediatrics [1]. While enriched in certain rare tumors, *NTRK* gene fusions are infrequent in more common cancers (often <1%) [2, 3]. Three *NTRK* genes (*NTRK1*, *NTRK2*, and *NTRK3*) respectively encode the transmembrane tropomyosin receptor kinase (TRK) A, B, and C proteins. TRK inhibitors are targeted drugs that block the activated kinase function of the wild-type or chimeric TRK fusion protein that results from the *NTRK* gene fusion.

There is a need to identify individuals with *NTRK* gene fusion tumors across real-world settings to describe the treatments they receive and their outcomes, and this can be met by linking patient genomic data to longitudinal electronic health records (EHRs). For example, in our recent clinicogenomic (pilot) study of patients with papillary thyroid cancer (PTC) [4], we demonstrated the feasibility of generating NGS data of tumor samples from the Auria Biobank in the Turku region of Finland linked at the patient level to hospital EHRs and vital statistics. This enabled detailed analyses of clinical cohorts of the sample donors defined by their tumor genome. PTC was selected for the pilot study due to its relatively high prevalence of *NTRK* gene fusions compared with other common cancer types [2, 5]. This present work expands our investigation to evaluate the feasibility of the same data sources to identify and evaluate patients with *NTRK* gene fusions in other solid tumors in adults and pediatrics. In adults, we selected colorectal cancer (CRC) and non-small cell lung cancer (NSCLC) due to their high global incidence despite a low frequency of *NTRK* gene fusion, and salivary gland cancer and sarcoma due to their low global incidence [6] combined with a relatively high frequency of *NTRK* gene fusions [2]. In pediatrics, we evaluated any type of solid tumor. The objectives of this present study were, firstly, to determine the frequency of *NTRK* gene fusions in these real-world patient populations, and, secondly, to describe the tumor, clinical, and other characteristics of patients positive for *NTRK* gene fusions.

## Materials and methods

### Study design and data source

This was a population-based clinicogenomic study set in the Turku region of Finland. Auria Biobank stores human biological samples and related healthcare data from the area of southwest Finland based on the donor’s consent or legal transfer to the biobank according to the Finnish Biobank Act. The FFPE tumor samples from donors treated and operated in Turku University Hospital are collected and included in the biobank, and can be used for research after their clinical use in the Department of Pathology. The samples are linkable to Turku University Hospital’s EHRs at the individual patient level with comprehensive data coverage since 2004. Further details of the Auria Biobank and linked data sources – hospital EHRs and vital statistics records in Turku University Hospital – have been described previously [4]. For this present study, use of patient data, including cancer tumor samples, was approved by Auria Biobank’s Scientific Steering Committee (Decisions AB18-6900, AB18-2303 and AB18-9957), Hospital District of Southwest Finland (research permission T278/2018), and by Statistics Finland (research permission TK-53-448-20).

### Study cohorts

Five study cohorts were identified: four comprised adults (≥ 18 years of age) with either CRC, NSCLC, salivary gland cancer, or sarcoma, and the fifth included pediatrics (< 18 years of age) with any type of solid tumor. FFPE samples for IHC analysis were initially selected at Auria Biobank based on the topography and pathologist’s diagnosis for the sample, whenever there was a tumor sample available and sufficient for research. To be included into the clinicogenomic part of the study, patients were required to have received a histologically-confirmed diagnosis of their cancer in the Hospital District of Southwest Finland between January 2005 and December 2019 (see Supplementary Figures 1–5).

### NTRK gene fusion identification and confirmation

For reasons of operational efficiency, we undertook a two-step process using IHC as a primary identification technique followed by orthogonal validation of fusion via NGS testing for confirmation [7]. Expression of TRK protein was determined by pan-TRK immunohistochemistry (IHC) staining of tumor samples using antibody clone EPR17341 (Abcam, Cambridge, MA, USA) [8] and OptiView DAB IHC detection kits on Ventana Discovery Ultra autostainers [8]. Note that here a laboratory developed test was used; while the antibody today is also part of an in vitro diagnostic [9] that was not yet available when this work was carried out. Stained slides were scored by a certified pathologist (KJ) from Dresden University Hospital within weeks after staining. Four categories were used to score the pan-TRK staining: ‘0’ for no staining, ‘1’ for weak, ‘2’ for moderate, and ‘3’ for strong. Further, the pathologist estimated the percentage of tumor and adjacent normal cells on each slide, and the tumor area was scored according to subcellular compartment (cytoplasmic, membrane, perinuclear, nuclear). For each compartment, the score of the predominant staining was recorded together with its percentage (e.g. 80% of tumor cytoplasm being stained moderately). Following this, the subset of samples flagged by IHC as pan-TRK positive (plus an arbitrary number of randomly selected IHC negative samples) was analyzed with next-generation sequencing (NGS) to confirm the result and determine the fusion partner, at the DIN EN ISO 15189:2013 certified clinical laboratory (Biopticka SRO, Plzen, Czech Republic), also employed as a central laboratory for *NTRK* NGS testing in Bayer’s clinical trials. The TruSight^TM^ Tumor 170 assay (TST170; Illumina, San Diego, CA, USA), which simultaneously analyzes DNA and RNA, was selected due to its comprehensive cover of 170 genes associated with solid tumors [4]. The DNA fraction is analyzed for single-nucleotide variants/indels and amplifications, and the RNA fraction for fusions/splice variants of 55 genes (including NTRK1/2/3) [9]. The Illumina TruSight 170 panel (TST170) is designed to target and enrich for fusions involving specific genes using hybrid capture technology. The advantage of this technology is that knowledge of only one of the partners is required, allowing for the potential discovery of novel fusion partners [10]. For example, others have used TST170 because it can detect known and unknown *ROS1* fusions [11, 12]. In our present study, fusion calling was performed using Illumina’s algorithm V2.0.1.8, as used previously [4]. The implementation of the assay at two clinical molecular diagnostics laboratories, according to AMP/CAP guidelines, has been described by others [13].

### Linkage to EHRs and follow-up

Through patient-level linkage to Turku University Hospital EHRs and vital statistics, we obtained data on patient demographics, comorbidities, lifestyle variables, laboratory test results, cancer treatments, and hospital visits, at the time of cancer diagnosis. The microsatellite instability (MSI) measurements of the adult CRC cohort members as part of patient care were collected from each patient’s EHR. MSI status was defined based on IHC testing for the following four DNA mismatch repair gene products: MLH1, MSH2, MSH6, PMS2. The sample was interpreted as MSS when a normal result was shown for all proteins, MSI-low when an abnormal result was shown for one of the four proteins, and MSI-high (MSI-H) when an abnormal result was shown for at least two of the four proteins. Patients were followed from 1 year before their cancer diagnosis until death, the end of their available observational period or the end of the study (December 2019) whichever came first.

### Statistical analysis

There were no pre-specified hypotheses; all data analysis was exploratory and descriptive. *NTRK* gene fusion frequency was expressed as a percentage of patients for whom this was NGS-confirmed (separately for each of the five cohorts) as well as a percentage of all patients whose tissue sample underwent IHC testing in each cohort. Characteristics of patients NGS confirmed as positive for *NTRK* gene fusion (including features of the tumor, treatment, and lifestyle characteristics) were described on an individual basis. However, to preserve patient privacy, sex and age at cancer diagnosis were not described for individual patients but were presented as overall frequency distributions and median values, respectively. Descriptive analyses were also performed for each cohort stratified by *NTRK* gene fusion status (i.e. positive or wild-type), with data summarized using frequency counts and percentages for categorical variables, and with medians and inter-quartile range for continuous variables. Analyses were undertaken using SAS version 9.4.

## Results

### NTRK gene fusion frequency

Among patients with tumors positive for TRK protein expression after IHC staining, the percentages confirmed as *NTRK* gene fusion positive after NGS were 80% (8/10) for CRC, 5% (1/21) for NSCLC, 6% (1/18) for salivary cancer, 0% (0/21) for sarcoma, and 25% (4/16) for pediatrics. Overall, the frequencies of *NTRK* gene fusion in adult tumors following confirmation by NGS were 0.7% (8/1,151) for CRC, 0.3% (1/288) for NSCLC, 0.9% (1/114) for salivary gland cancer, and 0% (0/379) for sarcoma, and in pediatric solid tumors it was 3.1% (4/127) (Table 1). Among randomly-selected IHC-negative samples (17 for CRC, 15 for NSCLC, 3 for salivary gland cancer, 3 for sarcoma, and 1 for pediatric solid tumors), all were NGS-confirmed as negative.

**Table 1:** *NTRK* gene fusion testing across selected solid tumors in adults, and solid tumor in pediatrics.

Tumor type	Number of patients	Number of samples submitted to IHC	Number of IHC-tested samples submitted to NGS*	Number of NGS-confirmed samples
**Adults**				
CRC	1,151	1,159	10	8
NSCLC	288	294	21	1
Sarcoma	379	381	21	0
Salivary	114	115	18	1
**Pediatrics**	127	149	16	4

CRC: colorectal cancer; IHC: immunohistochemistry; NGS: next-generation sequencing; NSCLC: non-small cell lung cancer; *NTRK*: neurotrophic tyrosine receptor kinase.

* Contains all IHC-positive samples. Additionally, further IHC-negative samples were randomly selected for NGS, all of which were found to be NTRK fusion negative. See Supplemental figures 1–5 for details.

### Characteristics of adults with confirmed NTRK gene fusion

Genomic and other characteristics of the 10 adults positive for *NTRK* gene fusion (eight CRC, one NSCLC, and one salivary gland cancer) are shown in Table 2. Of the eight patients with CRC, six had tumors harboring an *NTRK1* gene fusion, with the fusion partner being *TPM3* in three patients, and *TPR*, *LMNA,* and *IRF2BP2* in one patient each. Of the two other patients with CRC, one had a tumor harboring a *GABRG1::NTRK3* fusion, and the other had a tumor harboring the *NTRK2* gene with two different fusion partners identified from one sample *– FXN* and *LPPR1.* Five of these eight patients with CRC had tumors tested for MSI with four mismatch repair proteins (MLH1, MSH2, MSH6, and PMS2). Four of the five patients had abnormal (negative) IHC in MLH1 and PMS2 and were interpreted to represent MSI-H. The patient with NSCLC harbored an *SGCZ::NTRK3* fusion tumor, while the patient with salivary gland cancer harbored an *ETV6::NTRK3* fusion. Median age at cancer diagnosis for the 10 patients with *NTRK* gene fusion tumors was 67 years; seven were female. Four patients (three CRC, one NSCLC) received treatment with chemotherapy, while only one patient (with CRC) received treatment with radiotherapy; all underwent multiple procedures after cancer diagnosis. None was lost to follow-up, and all were alive at the end of their individual follow-up period (confirmed by data from Statistics Finland).

**Table 2:** Genomic, tumor, clinical, and lifestyle characteristics among adults with CRC (*N* = 8), NSCLC (*N* = 1), and salivary gland (*N* = 1) tumors harboring an *NTRK* gene fusion.

Characteristics	CRC								NSCLC	Salivary
	Patient 1	Patient 2	Patient 3	Patient 4	Patient 5	Patient 6	Patient 7	Patient 8	Patient 1	Patient 1
**Genomic characteristics**										
*NTRK* gene	*NTRK1*	*NTRK3*	*NTRK2*	*NTRK1*	*NTRK1*	*NTRK1*	*NTRK1*	*NTRK1*	*NTRK3*	*NTRK3*
*NTRK* gene fusion partner	*TPM3*	*GABRG1*	*FXN; LPPR1*	*TPM3*	*TPR*	*LMNA*	*TPM3*	*IRF2BP2*	*SGCZ*	*ETV6*
Other genomic co-alterations^*^	No	Yes	Yes	Yes	Yes	Yes	No	Yes	No	No
**Patient characteristics**										
Diagnosis year	2008	2009	2009	2015	2017	2018	2018	2019	2019	2006
BMI (kg/m^2^)	24	26	Unknown	29	23	23	25	29	39	Unknown
Smoking status	Past	Current	Past	Never	Never	Never	Never	Unknown	Current	Current
CCI^†^	Unknown	0	2	0	0	0	0	0	0	0
**Clinical characteristics**										
Zubrod score	1	1	1	Unknown	1	Unknown	Unknown	Unknown	1	Unknown
Grade	II	III	III	III	III	II	III	III	II	Unknown
Stage (AJCC)	IIIB	IIIC	IIB or IIC	II	IIA	Unknown	Unknown	Unknown	IVA	I
TNM	T3N1M0	pT4N2M0	pT4N0M0	T3N0M0	pT3N0	pT3N0	T3N0	T4aN0	T4N0M1b	T2N0M0
MSI status	MSS	Unknown	Unknown	MS-H	Unknown	MSI-H	MSI-H	MSI-H	NA	NA
Chemotherapy after diagnosis	Yes	Yes	No	No	Yes	No	No	No	Yes	No
Radiotherapy after diagnosis	Yes	No	No	No	No	No	No	No	No	No
**Survival status**										
Study follow-up time (years)	12	10	10	4	2	2	2	0	1	14
Deceased (as of Dec 31, 2019)	No	No	No	No	No	No	No	No	No	No

AJCC: American Joint Committee on Cancer; BMI: body mass index; CCI: Charlson Comorbidity Index; CRC: colorectal cancer; MSI-H: microsatellite instability high; MSS: microsatellite stable; NA: not applicable; NSCLC: non-small cell lung cancer; *NTRK*: neurotrophic tyrosine receptor kinase; TNM: tumor, node, metastasis.

* Other genomic co-alterations included in the TruSight™ Tumor 170 assay (Illumina, San Diego, CA, USA). To preserve patient confidentiality, individual-level age and sex data have been suppressed, and study follow-up time has been rounded.

† According to Quan et al. [14].

### Characteristics of pediatrics with confirmed NTRK gene fusion

Genomic and other characteristics of the four pediatric patients positive for *NTRK* gene fusion are shown in Table 3. There was one case each of fibrosarcoma, Ewing’s sarcoma, primitive neuroectodermal tumor, and PTC. The *NTRK* gene and fusion partner was *TPM3::NTRK1* (fibrosarcoma), *LPPR1::NTRK2* (Ewing’s sarcoma), *DAB2IP::NTRK2* (primitive neuroectodermal tumor), and *RAD51B::NTRK3* (PTC). The median age of the four patients was 10 years; there were 3 males and 1 female. The patient with Ewing’s sarcoma and the patient with primitive neuroectodermal tumor were still alive at the end of their individual follow-up (at 1 year both had received chemotherapy and radiotherapy). The patient with fibrosarcoma and the patient with PTC died within their individual observation periods (15 years and 7 years’ follow-up, respectively; neither had received chemotherapy nor radiotherapy); all pediatric patients underwent multiple procedures after cancer diagnosis.

**Table 3:** Genomic, tumor, clinical, and lifestyle characteristics among pediatrics harboring an *NTRK* gene fusion (*N* = 4).

Characteristics	Fibrosarcoma	Ewing’s sarcoma	Primitive neuroectodermal tumor	Papillary thyroid carcinoma
	Patient 1	Patient 2	Patient 3	Patient 4
**Genomic characteristics**				
*NTRK* gene	*NTRK1*	*NTRK2*	*NTRK2*	*NTRK3*
*NTRK* gene fusion partner	*TPM3*	*LPPR1*	*DAB2IP*	*RAD51B*
Other genomic co-alterations	No	Yes	Yes	Yes
**Patient characteristics**				
Diagnosis year	2004	2004	2006	2012
BMI (kg/m^2^)	Unknown	Unknown	Unknown	20
CCI^*^	2	2	Unknown	Unknown
**Clinical characteristics**				
Zubrod score	Unknown	Unknown	Unknown	1
Grade	Unknown	Unknown	III	Unknown
Stage (AJCC)	Unknown	IV	Unknown	Unknown
TNM	Unknown	Unknown	Unknown	T1N0
Chemotherapy after diagnosis	No	Yes	Yes	No
Radiotherapy after diagnosis	No	Yes	Yes	no
**Survival status**				
Study follow-up time (years)	15	1	1	7
Deceased (as of Dec 31, 2019)	No	Yes	Yes	No

AJCC: American Joint Committee on Cancer; BMI: body mass index; CCI: Charlson Comorbidity Index; *NTRK*: neurotrophic tyrosine receptor kinase; TNM: tumor, node, metastasis.

* According to Quan et al. [14].

To preserve patient confidentiality, individual-level age and sex data have been suppressed, and study follow-up time has been rounded.

### Comparison of patients with confirmed NTRK gene fusion vs. NTRK wild-type

Characteristics of the CRC, NSCLC, and salivary gland cancer cohorts according to the presence/absence of *NTRK* gene fusion tumors are shown in Table 4 (data for the sarcoma cohort are not shown due to all patients having *NTRK* fusion negative tumors). The data analysis was carried out for the study cohort diagnosed between 2005 and 2019 when the hospital EHR data was most complete. Among the CRC cohort, MSI was seen in a notably higher proportion of patients with an *NTRK* gene fusion tumor versus those with wild-type tumors (50.0% vs. 4.4%). Further, *NTRK*-positive tumors were commonly located on the right side (37.5% vs. 7.6%) and in either the ascending colon (25.0% vs. 2.1%) or transverse colon (25.0% vs. 2.0%). The eight CRC patients with an *NTRK* gene fusion tumor were, on average, slightly younger than patients with wild-type tumors (*N* = 1,080) and were more frequently female and non-obese. Characteristics of the pediatric cohort according to the presence/absence of an *NTRK* gene fusion tumor are shown in Table 5; patients harboring an *NTRK* gene fusion tumor were, on average, younger than those with a *NTRK* wild-type tumor. Data for all four *NTRK* positive cases (diagnosed in 2004, 2004, 2006 and 2012) were compared to the data for *NTRK* wild-type cases diagnosed between 2005 and 2019.

**Table 4:** Characteristics of the CRC, NSCLC, and salivary gland cancer cohorts of adults with solid tumors (*N* = 1088) according to presence/absence of *NTRK* gene fusion.

	CRC		NSCLC		Salivary gland cancer	
Characteristic	*NTRK* gene fusion *N* = 8	*NTRK* wild-type *N* = 1080	*NTRK* gene fusion *N* = 1	*NTRK* wild-type *N* = 255	*NTRK* gene fusion *N* = 1	*NTRK* wild-type *N* = 58
**Age at CRC diagnosis**						
Median (IQR)	67.5 (65.0–72.5)	68.9 (61.0–75.7)	68.3 (NA)	67.8 (62.6–72.3)	66.4 (NA)	66.4 (60.7–76.7)
18–59	1 (12.5)	237 (21.9)	0 (0)	45 (17.6)	0 (0)	11 (19.0)
60–69	4 (50.0)	367 (34.0)	1 (100)	117 (45.9)	1 (100)	25 (43.1)
70–79	3 (37.5)	353 (32.7)	0 (0)	83 (32.5)	0 (0)	13 (22.4)
≥80	0 (0)	123 (11.4)	0 (0)	10 (3.9)	0 (0)	9 (15.5)
**Sex**						
Female	6 (75.0)	513 (47.5)	1 (100)	121 (47.5)	0 (0)	40 (69.0)
Male	2 (25.0)	567 (52.5)	0 (0)	134 (52.5)	1 (100)	18 (31.0)
**BMI, kg/m** ^2^						
< 30 (non-obese)	7 (87.5)	735 (68.1)	0 (0)	164 (64.3)	0 (0)	23 (39.7)
≥ 30 (obese)	0 (0.0)	191 (17.7)	1 (100)	54 (21.2)	0 (0)	10 (17.2)
Missing	1 (12.5)	154 (14.3)	0 (0)	37 (14.5)	1 (100)	25 (43.1)
**Smoking status**						
Current	1 (12.5)	137 (12.7)	1 (100)	134 (52.5)	1 (100)	8 (13.8)
Former	2 (25.0)	205 (19.0)	0 (0)	72 (28.2)	0 (0)	10 (17.2)
Never	4 (50.0)	399 (36.9)	0 (0)	46 (18.0)	0 (0)	24 (41.4)
Missing	1 (12.5)	339 (31.4)	0 (0)	3 (1.2)	0 (0)	16 (27.6)
**Charlson Comorbidity Index at diagnosis**						
0	6 (75.0)	754 (69.8)	1 (100)	68 (26.7)	1 (100)	16 (27.6)
1	0 (0)	83 (7.7)	0 (0)	58 (22.7)	0 (0)	4 (6.9)
2	1 (12.5)	76 (7.0)	0 (0)	47 (18.4)	0 (0)	5 (8.6)
≥3	0 (0.0)	49 (4.5)	0 (0)	19 (7.5)	0 (0)	1 (1.7)
Missing	1 (12.5)	118 (10.9)	0 (0)	63 (24.7)	0 (0)	32 (55.2)
**Zubrod scores**						
0	0 (0)	153 (14.2)	0 (0)	66 (25.9)	0 (0)	2 (3.4)
0–1	0 (0)	19 (1.8)	0 (0)	8 (3.1)	0 (0)	–
1	4 (50.0)	388 (35.9)	1 (100)	91 (35.7)	0 (0)	22 (37.9)
1–2	0 (0)	42 (3.9)	0 (0)	2 (0.8)	0 (0)	–
2	0 (0)	6 (0.6)	0 (0)	12 (4.7)	0 (0)	1 (1.7)
2–3	0 (0)	3 (0.3)	0 (0)	1 (0.4)	0 (0)	2 (3.4)
3	0 (0)	–	0 (0)	1 (0.4)	0 (0)	–
4	0 (0)	1 (<0.1)	0 (0)	1 (0.4)	0 (0)	–
Missing	4 (50.0)	440 (40.7)	0 (0)	73 (28.6)	1 (100)	31 (53.4)
**Cancer stage at diagnosis, AJCC** ^*^						
I	0 (0)	42 (3.9)	0 (0)	34 (13.3)	0 (0)	0 (0)
II	2 (25.0)	114 (10.6)	0 (0)	15 (5.9)	0 (0)	1 (1.7)
III	1 (12.5)	114 (10.6)	0 (0)	18 (7.1)	0 (0)	0 (0)
IV	0 (0)	11 (1.0)	1 (100)	8 (3.1)	0 (0)	0 (0)
Missing	5 (62.5)	799 (74.0)	0 (0)	180 (70.6)	1 (100)	57 (98.3)
**Tumor location side**						
Left	1 (12.5)	666 (61.7)	0 (0)	0 (0)	0 (0)	0 (0)
Right	3 (37.5)	82 (7.6)	0 (0)	0 (0)	0 (0)	0 (0)
Unclear	2 (25.0)	22 (2.0)	0 (0)	0 (0)	0 (0)	0 (0)
Missing	2 (25.0)	310 (28.7)	1 (100)	25 (100)	1 (100)	58 (100)
**Microsatellite instability**						
Yes	4 (50.0)	47 (4.4)	NA	NA	NA	NA
No	1 (12.5)	237 (21.9)	NA	NA	NA	NA
Unclear	0 (0)	4 (0.4)	NA	NA	NA	NA
Missing	3 (37.5)	792 (73.3)	1 (100)	255 (100)	1 (100)	58 (100)
**Cancer treatments**						
Radiotherapy	1 (12.5)	323 (29.9)	0 (0)	72 (28.2)	0 (0)	32 (55.2)
Chemotherapy	3 (37.5)	492 (45.6)	1 (100)	117 (45.9)	0 (0)	53 (91.4)
**Procedures,**^†^ **median (IQR)**						
Before cancer diagnosis	8.5 (4.5–17.0)	8.0 (3.0–18.0	18.0 (NA)	17.0 (9.0–35.0)	8.0 (NA)	5.5 (3.0–17.0)
After cancer diagnosis	14.5 (5.0–46.0)	26.0 (14.0–47.0)	28 (NA)	33.0 (2.0–55.0)	8.0 (NA)	24.0 (9.0–53.0)
**Hospitalizations, mean number per patient (SD)**						
Visit	60.4 (72.2)	48.0 (57.3)	20.0 (NA)	46.9 (39.4)	20.0 (NA)	74.7 (129.4)
Ward	4.0 (4.6)	4.2 (4.0)	5.0 (NA)	3.0 (3.4)	2.0 (NA)	3.0 (3.6)
**Deaths/survival**						
Length of observation while alive (median, IQR)	3.2 (1.9–10.3)	6.7 (2.5–10.0)	0.61 (NA)	3.86 (1.48–8.01)	14.0 (NA)	7.0 (2.5–11.5)
Deaths within 5 years	0 (0)	33 (3.1)	0 (0)	42 (16.5)	0 (0)	11 (19.0)
Death within 10 years	0 (0)	67 (6.2)	0 (0)	52 (20.4)	0 (0)	15 (25.9)
Total deaths (as of 31 Dec 2019)	0 (0)	91 (8.4)	0 (0)	58 (22.7)	0 (0)	18 (31.0)

Data are n (%) or median (IQR), or mean (SD) as appropriate.

AJCC: American Joint Committee on Cancer; BMI: body mass index; CRC: colorectal cancer; IQR: inter-quartile range; NSCLC: non-small cell lung cancer; NA: not applicable; *NTRK*: neurotrophic tyrosine receptor kinase; SD: standard deviation.

Note, a dash in the table cells indicates that data was missing. NA means that the variables were not evaluated.

* AJCC stage was derived based on the available TNM data.

† Included extensive surgical operations, small operations, medical imaging, device-assisted examinations, and some therapies.

‘Missing’ cancer stage classification is due to missing or incomplete TNM.

**Table 5:** Characteristics of the pediatric cohort with solid tumors (*N* = 70) according to presence/absence of *NTRK* gene fusion.

Characteristic	*NTRK* gene fusion *N* = 4	*NTRK* wild-type *N* = 66
**Age at cancer diagnosis**		
Median (IQR)	9.9 (5.4–13.6)	10.8 (4.6–15.0)
<1	0 (0.0)	8 (12.1)
1–4	1 (25.0)	9 (13.6)
5–9	1 (25.0)	15 (22.7)
10–17	2 (50.0)	34 (51.5)
**Sex**		
Female	1 (25.0)	31 (47.0)
Male	3 (75.0)	35 (53.0)
**BMI, kg/m** ^2^		
<30 (non-obese)	1 (25.0)	44 (66.7)
≥30 (obese)	0 (0.0)	2 (3.0)
Missing	3 (75.0)	20 (30.3)
**Smoking status**		
Current	0 (0.0)	3 (4.5)
Former	0 (0.0)	0 (0.0)
Never	1 (25.0)	11 (16.7)
Missing	3 (75.0)	52 (78.8)
**Charlson Comorbidity Index at diagnosis**		
0	0 (0.0)	34 (51.5)
1	0 (0.0)	2 (3.0)
2	2 (50.0)	3 (4.5)
≥3	0 (0.0)	1 (1.5)
Missing	2 (50.0)	26 (39.4)
**Zubrod scores**		
0	0 (0.0)	7 (10.6)
0–1	0 (0.0)	0 (0.0)
1	1 (25.0)	6 (9.1)
1–0	0 (0.0)	0 (0.0)
1–2	0 (0.0)	0 (0.0)
2	0 (0.0)	0 (0.0)
2–3	0 (0.0)	0 (0.0)
3	0 (0.0)	0 (0.0)
4	0 (0.0)	0 (0.0)
Missing	3 (75.0)	53 (80.3)
**Cancer stage at diagnosis, AJCC** ^*^		
I	0 (0.0)	1 (1.5)
II	0 (0.0)	4 (6.1)
III	0 (0.0)	3 (4.5)
IV	1 (25.0)	8 (12.1)
Missing	3 (75.0)	50 (75.8)
**Cancer treatments**		
Radiotherapy	2 (50.0)	25 (37.9)
Chemotherapy	2 (50.0)	42 (63.6)
**Procedures,**^†^ **median (IQR)**		
Before cancer diagnosis	12.5 (9.0–14.5)	3.0 (0.0–5.0)
After cancer diagnosis	28.0 (17.0–44.5)	58.0 (22.0–109.0)
**Hospitalizations, mean number per patient (SD)**		
Visit	66.5 (34.9)	124.0 (102.0)
Ward	12.0 (12.3)	16.9 (14.6)
**Deaths/survival**		
Length of observation while alive (median, IQR)	4.3 (1.0–11.3)	8.6 (6.7–12.1)
Deaths within 5 years	2 (50.0)	14 (21.2)
Death within 10 years	2 (50.0)	16 (24.2)
Total deaths (as of 31 Dec 2019)	2 (50.0)	16 (24.2)

Data are n (%) or median (IQR), or mean (SD) as appropriate.

AJCC: American Joint Committee on Cancer; BMI: body mass index; IQR: inter-quartile range; NSCLC: non-small cell lung cancer; *NTRK*: neurotrophic tyrosine receptor kinase; SD: standard deviation.

* AJCC stage was derived based on the available TNM data. Unknown classification is due to missing or incomplete TNM.

† Included extensive surgical operations, small operations, medical imaging, device-assisted examinations, and some therapies.

## Discussion

This population-based clinicogenomic study builds on our initial work on *NTRK* gene fusions in PTC [4], and previous work on other biomarkers in oncology [15, 16], to further support the utility of linking patient-level genomic data from the Auria Biobank to longitudinal EHRs and vital statistics. The infrequency of *NTRK* gene fusions seen in adults and pediatrics with solid tumors (0.7% for CRC, 0.3% for NSCLC, 0% for sarcoma, and 3.1% for pediatrics) are mostly in line with expectations from the literature of their low prevalence among adult tumors [2, 3, 5, 17–20] and further support the higher prevalence among tumors in pediatrics versus adults [2, 3, 21] (see also the Supplementary Table for the technologies used for fusion detection in these cited studies). Among the 379 patients with sarcomas, none had an *NTRK* gene fusion tumor; thus the frequency was lower than expected from the literature (approx. 0.2–0.8%). Since our work – carried out in 2018/2019 – others have noted the poor performance of this antibody in IHC of sarcomas [5].

The high prevalence of MSI-H in patients with CRC harboring an *NTRK* gene fusion tumor is consistent with previous research [2, 18, 22–24]. In line with previous research, our findings also show *NTRK* gene fusions in patients with CRC occur mostly in right-sided tumors [22, 25, 26] and are located in the ascending or transcending colon [2]. Three of the fusion partners among adults with CRC harboring an *NTRK* gene fusion tumor have been commonly reported in the literature, including *TPM3::NTRK1* [8, 17, 20, 24, 27–30], *TPR::NTRK1* [20, 24, 25, 29, 30], and *LMNA::NTRK1* [8, 20, 24, 25, 27, 28, 30, 31]. The other *NTRK* gene fusions that we identified in our adult CRC cohort were *IRF2BP2::NTRK1* (which has been reported previously among adult tumors) [2], *GABRG1::NTRK3*, and a fusion of the *NTRK2* gene with two different fusion partners – *FXM* and *LPPR1* – identified from a single tissue sample. We did not identify any patients with an *ELM4::NTRK3* gene fusion tumor as previously found by others [20, 27, 30, 32]. The *ETV6::NTRK3* fusion – detected in a single patient with a salivary gland tumor – has been commonly reported by others [31, 33–36]. However, other reports of the *SGCZ::NTRK3* fusion, which we detected in a single patient with NSCLC, are lacking. Conversely, several *NTRK* gene fusions, previously documented in patients with NSCLC, were not found in our NSCLC cohort, including *TPM3::NTRK1* [8, 17], *SQSTM1* partnered with *NTRK1/NTRK2/NTRK3* [20, 28, 37, 38], *ETV6-NTRK3* [17], *IRF2BP2-NTRK1* [8], *EPS15::NTRK1* [37], and *CD74-NTRK1* [28]. Furthermore, no patients in our sarcoma cohort harbored an *NTRK* gene fusion tumor, yet they have been reported in the literature by others [8, 17, 18, 28, 31]. Of the *NTRK* fusion partners we identified in pediatrics, *TPM3::NTRK1* (fibrosarcoma) has been documented in pediatrics by others [39, 40], and *DAB2IP::NTRK2* (primitive neuroectodermal tumor) has been previously documented in adults. The other two *NTRK* gene fusions in pediatrics were an *LPP1::NTRK2* fusion (Ewing’s sarcoma), and a *RAD51B::NTRK3* (PTC); we did not identify *ETV6::NTRK3* [21, 41, 42, 43], *TPR::NTRK1* [41, 42] – *NTRK* gene fusions were more commonly reported among pediatrics with solid tumors.

The availability of TRK inhibitors as a targeted therapy for patients with an *NTRK* gene fusion tumor has enabled physicians to optimize treatment strategies in these patients, with the potential to improve outcomes and quality of life [44–47]. As this was a descriptive study and no statistical comparisons were made between *NTRK* fusion positive versus negative patients, these results cannot infer the prognostic value of *NTRK* fusion. Furthermore, the small sample size of the *NTRK* fusion positive patients meant that it would not be possible to draw any meaningful conclusions from any survival analysis undertaken. Some studies have suggested an unclear prognostic significance of *NTRK* fusions [21, 48], while others have suggested *NTRK* fusions could be a negative prognostic factor of survival [49–51]. Nevertheless, our study demonstrates the feasibility of using the Auria Biobank and linked data sources to do so in future. A strength of our study was the wide range of patient data enabling the study of a variety of patients and tumor characteristics, and the ability to follow them observationally. Additionally, the population-based sample was drawn from southwest Finland where Turku university hospital provides cancer care, and which has minimal migration between the other counties of Finland in all age groups apart from students and young adults [52]. Limitations of our study should also be acknowledged. Firstly, the lack of NGS testing for most tumor samples meant that in contrast to our previous related work in PTC [4], we were unable to calculate the accuracy of our IHC assay – neither sensitivity nor specificity could be determined. However, while we cannot rule out false negative IHC results, all randomly selected IHC-negative samples were confirmed as negative following NGS. Further, the presence of false positive IHC results might indicate that the threshold of IHC results was set appropriately low (i.e. was sufficiently sensitive) to subject any potential fusion positive sample to NGS. Indeed, the IHC result threshold for NGS testing was set for highest sensitivity, at the expense of some false positives (i.e. lower specificity). The two-step process used to identify *NTRK* gene fusions in this study is just one of several methods available, each differing with regards to sensitivity and specificity; global consensus on best diagnostic practices is emerging [7]. Secondly, there is the possibility of selection bias if there were any systematic differences between patients who had not provided consent for their tissue samples to be used for research purposes and those who had provided consent. Thirdly, missing EHR data on some patient characteristics/management limited a more complete understanding of the patient journey. Data were also unavailable on MSI testing in the pediatric cohort; this was a very heterogeneous cohort in terms of tumor type and the number of pediatric cases of CRC was very small. Fourthly, the limited size of the study cohorts may have led to estimates less precise than those reported from larger studies and could have been the reason for the lack of *NTRK* gene fusion tumors among the sarcoma cohort. Also, as only one patient each in the NSCLC and salivary cancer cohorts, and none in the sarcoma cohort were identified as harboring an *NTRK* gene fusion, this prevented comparisons between members of the respective cohort with *NTRK* wild-type tumors. Lastly our findings may not be generalizable to patients with *NTRK* gene fusion from other geographical areas.

In conclusion, our findings demonstrate the ability to perform a population-based clinicogenomic study using linked real-world data sources in Finland to identify and evaluate patients harboring an *NTRK* gene fusion. This work also supports previous research regarding the infrequent prevalence of these gene fusions in adult and pediatric solid tumors.

## Supplementary Material

Neurotrophic tyrosine receptor kinase gene fusions in adult and pediatric patients with solid tumors: a clinicogenomic biobank and record linkage study of expression frequency and patient characteristics from Finland

## Data Availability

The data that support the findings of this study are governed by Section 27 of Finland’s Biobank Act (2012), which regulates access to patient samples and health information. Further details regarding data availability can be obtained from Merja Perälä (author of this manuscript) of the Auria Biobank, University of Turku and Turku University Hospital; email merja.perala@tyks.fi

## References

[1] Cocco E, Scaltriti M, Drilon A. NTRK fusion-positive cancers and TRK inhibitor therapy. Nat Rev Clin Oncol. 2018 Dec;15(12):731–47. 10.1038/s41571-018-0113-0 30333516 PMC6419506

[2] Westphalen CB, Krebs MG, Le Tourneau C, et al. Genomic context of NTRK1/2/3 fusion-positive tumours from a large real-world population. NPJ Precis Oncol. 2021 Jul 20;5(1):69. 10.1038/s41698-021-00206-y 34285332 PMC8292342

[3] O’Haire S, Franchini F, Kang Y-J, et al. Systematic review of NTRK 1/2/3 fusion prevalence pan-cancer and across solid tumours. Sci Rep. 2023;13(1):4116. 10.1038/s41598-023-31055-3 36914665 PMC10011574

[4] Zhang W, Schmitz AA, Kallionpää RE, et al. Neurotrophic-tyrosine receptor kinase gene fusion in papillary thyroid cancer: a clinicogenomic biobank and record linkage study from Finland. Oncotarget. 2024 Feb 5;15:106–16. 10.18632/oncotarget.28555 38329731 PMC10852057

[5] Solomon JP, Linkov I, Rosado A, et al. NTRK fusion detection across multiple assays and 33,997 cases: diagnostic implications and pitfalls. Modern Pathol. 2020;33(1):38–46. 10.1038/s41379-019-0324-7 PMC7437403 31375766

[6] Ferlay J, Ervik M, Lam F, et al. *Global cancer observatory: cancer today* [Internet]. Lyon: International Agency for Research on Cancer. [cited 24-04-2024]. Available from: https://gco.iarc.fr/today

[7] Yoshino T, Pentheroudakis G, Mishima S, et al. JSCO-ESMO-ASCO-JSMO-TOS: international expert consensus recommendations for tumour-agnostic treatments in patients with solid tumours with microsatellite instability or NTRK fusions. Ann Oncol. 2020 Jul;31(7):861–72. 10.1016/j.annonc.2020.03.299 32272210

[8] Hechtman JF, Benayed R, Hyman DM, et al. Pan-Trk immunohistochemistry is an efficient and reliable screen for the detection of NTRK fusions. Am J Surg Pathol. 2017 Nov;41(11):1547–51. 10.1097/PAS.0000000000000911 28719467 PMC5636652

[9] Roche Diagnostics. *VENTANA pan-TRK (EPR17341 Assay)* [Internet]. [cited 24-04-2024]. Available from: https://diagnostics.roche.com/global/en/products/lab/pan-trk-epr17341-assay-ventana-rtd001230.html

[10] Wong D, Yip S, Sorensen PH. Methods for identifying patients with tropomyosin receptor kinase (TRK) fusion cancer. Pathol Oncol Res. 2020;26(3):1385–99. 10.1007/s12253-019-00685-2 31256325 PMC7297824

[11] Heydt C, Ruesseler V, Pappesch R, et al. Comparison of in situ and extraction-based methods for the detection of ROS1 rearrangements in solid tumors. J Mol Diagn. 2019;21(6):971–84. 10.1016/j.jmoldx.2019.06.006 31382035

[12] [cited 24-04-2024]. Available from: https://www.illumina.com/content/dam/illumina-marketing/documents/products/datasheets/trusight-tumor-170-data-sheet-1170-2016-017.pdf

[13] Boyle TA, Mondal AK, Saeed-Vafa D, et al. Guideline-adherent clinical validation of a comprehensive 170-gene DNA/RNA panel for determination of small variants, copy number variations, splice variants, and fusions on a next-generation sequencing platform in the CLIA setting. Front Genet. 2021;12:503830. 10.3389/fgene.2021.503830 34093633 PMC8172991

[14] Quan H, Sundararajan V, Halfon P, et al. Coding algorithms for defining comorbidities in ICD-9-CM and ICD-10 administrative data. Medical Care. 2005;43(11):1130–9. 10.1097/01.mlr.0000182534.19832.83 16224307

[15] Vizcaya D, Farahmand B, Walter AO, et al. Prognosis of patients with malignant mesothelioma by expression of programmed cell death 1 ligand 1 and mesothelin in a contemporary cohort in Finland. Cancer Treat Res Commun. 2020;25:100260. 10.1016/j.ctarc.2020.100260 33310366

[16] Khan M, Khaznadar SS, Routila J, et al. Hepatocyte growth factor receptor overexpression predicts reduced survival but its targeting is not effective in unselected HNSCC patients. Head Neck. 2020 Apr;42(4):625–35. 10.1002/hed.26049 31919967

[17] Gatalica Z, Xiu J, Swensen J, et al. Molecular characterization of cancers with *NTRK* gene fusions. Mod Pathol. 2019;32(1):147–53. 10.1038/s41379-018-0118-3 30171197

[18] Bridgewater J, Jiao X, Parimi M, et al. Prognosis and oncogenomic profiling of patients with tropomyosin receptor kinase fusion cancer in the 100,000 genomes project. Cancer Treat Res Commun. 2022;33:100623. 10.1016/j.ctarc.2022.100623 36041373

[19] Okamura R, Boichard A, Kato S, et al. Analysis of NTRK alterations in pan-cancer adult and pediatric malignancies: implications for NTRK-targeted therapeutics. JCO Precis Oncol. 2018;2018:PO.18.00183. 10.1200/PO.18.00183 30637364 PMC6329466

[20] Marchetti A, Ferro B, Pasciuto MP, et al. NTRK gene fusions in solid tumors: agnostic relevance, prevalence and diagnostic strategies. Pathologica. 2022 Jun;114(3):199–216. 10.32074/1591-951X-787 35775706 PMC9248239

[21] Zhao X, Kotch C, Fox E, et al. NTRK fusions identified in pediatric tumors: the frequency, fusion partners, and clinical outcome. JCO Precis Oncol. 2021;1:PO.20.00250. 34036219 10.1200/PO.20.00250 PMC8140782

[22] Pietrantonio F, Di Nicolantonio F, Schrock AB, et al. ALK, ROS1, and NTRK Rearrangements in Metastatic Colorectal Cancer. J Natl Cancer Inst. 2017 Dec 1;109(12). doi: 10.1093/jnci/djx089. PMID: 29370427. 10.1093/jnci/djx089 29370427

[23] Cocco E, Benhamida J, Middha S, et al. Colorectal carcinomas containing hypermethylated MLH1 promoter and wild-type BRAF/KRAS are enriched for targetable kinase fusions. Cancer Res. 2019 Mar 15;79(6):1047–53. 10.1158/0008-5472.CAN-18-3126 30643016 PMC6420871

[24] Wang H, Li ZW, Ou Q, et al. NTRK fusion positive colorectal cancer is a unique subset of CRC with high TMB and microsatellite instability. Cancer Med. 2022 Jul;11(13):2541–9. 10.1002/cam4.4561 35506567 PMC9249987

[25] Chou A, Fraser T, Ahadi M, et al. NTRK gene rearrangements are highly enriched in MLH1/PMS2 deficient, BRAF wild-type colorectal carcinomas-a study of 4569 cases. Mod Pathol. 2020 May;33(5):924–32. 10.1038/s41379-019-0417-3 31792356

[26] Wu G, Diaz AK, Paugh BS, et al. The genomic landscape of diffuse intrinsic pontine glioma and pediatric non-brainstem high-grade glioma. Nat Genet. 2014;46(5):444–50. 10.1038/ng.2938 24705251 PMC4056452

[27] Wu S, Liu Y, Shi X, et al. Elaboration of NTRK-rearranged colorectal cancer: integration of immunoreactivity pattern, cytogenetic identity, and rearrangement variant. Digest Liver Dis. 2023;55(12):1757–64. 10.1016/j.dld.2023.04.019 37142453

[28] Bang H, Lee MS, Sung M, et al. NTRK fusions in 1113 solid tumors in a single institution. Diagnostics (Basel). 2022 Jun 13;12(6):1450. 10.3390/diagnostics12061450 35741260 PMC9222038

[29] Yamashiro Y, Kurihara T, Hayashi T, et al. NTRK fusion in Japanese colorectal adenocarcinomas. Sci Rep. 2021 Mar 11;11(1):5635. 10.1038/s41598-021-85075-y 33707574 PMC7952565

[30] Lasota J, Chłopek M, Lamoureux J, et al. Colonic adenocarcinomas harboring NTRK fusion genes: a clinicopathologic and molecular genetic study of 16 cases and review of the literature. Am J Surg Pathol. 2020 Feb;44(2):162–73. 10.1097/PAS.0000000000001377 31567189 PMC8170835

[31] Silvertown JD, Lisle C, Semenuk L, et al. Prevalence of NTRK fusions in Canadian solid tumour cancer patients. Mol Diagn Ther. 2023 Jan;27(1):87–103. 10.1007/s40291-022-00617-y 36194351 PMC9531629

[32] ARCHER Quiver Fusion Database [Internet]. [cited 24-04-2024]. Available from: http://quiver.archerdx.com

[33] Yokota T, Yukino H, Doi M, et al. Real-world experience of tropomyosin receptor kinase inhibition with entrectinib in ETV6-NTRK3 positive metastatic salivary secretory carcinoma: a case series. Head Neck. 2023 May;45(5):E10–15. 10.1002/hed.27346 36924196

[34] Florou V, Nevala-Plagemann C, Whisenant J, et al. Clinical activity of selitrectinib in a patient with mammary analogue secretory carcinoma of the parotid gland with secondary resistance to entrectinib. J Natl Compr Canc Netw. 2021 May;19(5):478–82. 10.6004/jnccn.2021.7022 34030125

[35] Rudzinski ER, Hechtman J, Roy-Chowdhuri S, et al. Diagnostic testing approaches for the identification of patients with TRK fusion cancer prior to enrollment in clinical trials investigating larotrectinib. Cancer Genet. 2022;260–261:46–52. 10.1016/j.cancergen.2021.11.006 34929613

[36] Wagner F, Greim R, Krebs K, et al. Characterization of an ETV6-NTRK3 rearrangement with unusual, but highly significant FISH signal pattern in a secretory carcinoma of the salivary gland: a case report. Diagn Pathol. 2021 Aug 9;16(1):73. 10.1186/s13000-021-01133-z 34372873 PMC8353763

[37] Overbeck TR, Reiffert A, Schmitz K, et al. NTRK gene fusions in non-small-cell lung cancer: real-world screening data of 1068 unselected patients. Cancers (Basel). 2023 May 29;15(11):2966. 10.3390/cancers15112966 37296928 PMC10252111

[38] Farago AF, Taylor MS, Doebele RC, et al. Clinicopathologic features of non–small-cell lung cancer harboring an NTRK gene fusion. JCO Precis Oncol. 2018;2018:PO.18.00037. 30215037 10.1200/PO.18.00037 PMC6132056

[39] Huson SM, Staab T, Pereira M, et al. Infantile fibrosarcoma with TPM3-NTRK1 fusion in a boy with bloom syndrome. Fam Cancer. 2022;21(1):85–90. 10.1007/s10689-020-00221-1 33219493 PMC8799568

[40] Pehlivan KC, Malicki DM, Levy ML, et al. TPM3-NTRK1 fusion in a pleomorphic xanthoastrocytoma presenting with haemorrhage in a child. BMJ Case Rep. 2020 Mar 12;13(3):e234347. 10.1136/bcr-2020-234347 PMC7069298 32169993

[41] Prasad ML, Vyas M, Horne MJ, et al. NTRK fusion oncogenes in pediatric papillary thyroid carcinoma in northeast United States. Cancer. 2016 Apr 1;122(7):1097–107. 10.1002/cncr.29887 26784937

[42] Ricarte-Filho JC, Li S, Garcia-Rendueles ME, et al. Identification of kinase fusion oncogenes in post-Chernobyl radiation-induced thyroid cancers. J Clin Invest. 2013 Nov;123(11):4935–44. 10.1172/JCI69766 24135138 PMC3809792

[43] Davis JL, Lockwood CM, Stohr B, et al. Expanding the spectrum of pediatric NTRK-rearranged mesenchymal tumors. Am J Surg Pathol. 2019 Apr;43(4):435–45. 10.1097/PAS.0000000000001203 30585824

[44] Waguespack SG, Drilon A, Lin JJ, et al. Efficacy and safety of larotrectinib in patients with TRK fusion-positive thyroid carcinoma. Eur J Endocrinol. 2022 Apr 29;186(6):631–43. 10.1530/EJE-21-1259 35333737 PMC9066591

[45] Demetri GD, De Braud F, Drilon A, et al. Updated integrated analysis of the efficacy and safety of entrectinib in patients with NTRK fusion-positive solid tumors. Clin Cancer Res. 2022;28(7):1302–12. 10.1158/1078-0432.CCR-21-3597 35144967 PMC9365368

[46] Bokemeyer C, Paracha N, Lassen U, et al. Survival outcomes of patients with tropomyosin receptor kinase fusion-positive cancer receiving larotrectinib versus standard of care: a matching-adjusted indirect comparison using real-world data. JCO Precis Oncol. 2023 Jan;7:e2200436. 10.1200/PO.22.00436 36689698 PMC9928633

[47] Kummar S, Berlin J, Mascarenhas L, et al. Quality of life in adult and pediatric patients with tropomyosin receptor kinase fusion cancer receiving larotrectinib. Curr Probl Cancer. 2021 Dec;45(6):100734. 10.1016/j.currproblcancer.2021.100734 33865615

[48] Bazhenova L, Lokker A, Snider J, et al. TRK fusion cancer: patient characteristics and survival analysis in the real-world setting. Target Oncol. 2021;16(3):389–99. 10.1007/s11523-021-00815-4 33893941 PMC8105201

[49] Hibar DP, Demetri GD, Peters S, et al. Real-world survival outcomes in patients with locally advanced or metastatic NTRK fusion-positive solid tumors receiving standard-of-care therapies other than targeted TRK inhibitors. PLoS One. 2022;17(8):e0270571. 10.1371/journal.pone.0270571 35939431 PMC9359555

[50] Lassen U, Bokemeyer C, Garcia-Foncillas J, et al. Prognostic value of neurotrophic tyrosine receptor kinase gene fusions in solid tumors for overall survival: a systematic review and meta-analysis. JCO Precis Oncol. 2023 Jun;7:e2200651. 10.1200/PO.22.00651 37384865 PMC10581655

[51] Manea CA, Badiu DC, Ploscaru IC, et al. A review of NTRK fusions in cancer. Ann Med Surg. 2022;79:103893. 10.1016/j.amsu.2022.103893 PMC9289232 35860155

[52] Statistics Finland [Internet]. [cited 24-04-2024]. Available from: https://pxdata.stat.fi:443/PxWeb/sq/f784d3b8-2081-4b52-b09f-cd98b032cb43

