# Supplementary material for: Neurotrophic tyrosine receptor kinase gene fusions in adult and pediatric patients with solid tumors: a clinicogenomic biobank and record linkage study of expression frequency and patient characteristics from Finland
Source: Acta Oncol. 2024 Jul 5;63:26452. doi: 10.2340/1651-226X.2024.26452 (PMC11332464; doi:10.2340/1651-226X.2024.26452)
Supplement: Neurotrophic tyrosine receptor kinase gene fusions in adult and pediatric patients with solid tumors: a clinicogenomic biobank and record linkage study of expression frequency and patient characteristics from Finland [file AO-63-26452-s1.pdf]

Supplementary material has been published as submitted. It has not been copyedited, or typeset by Acta Oncologica

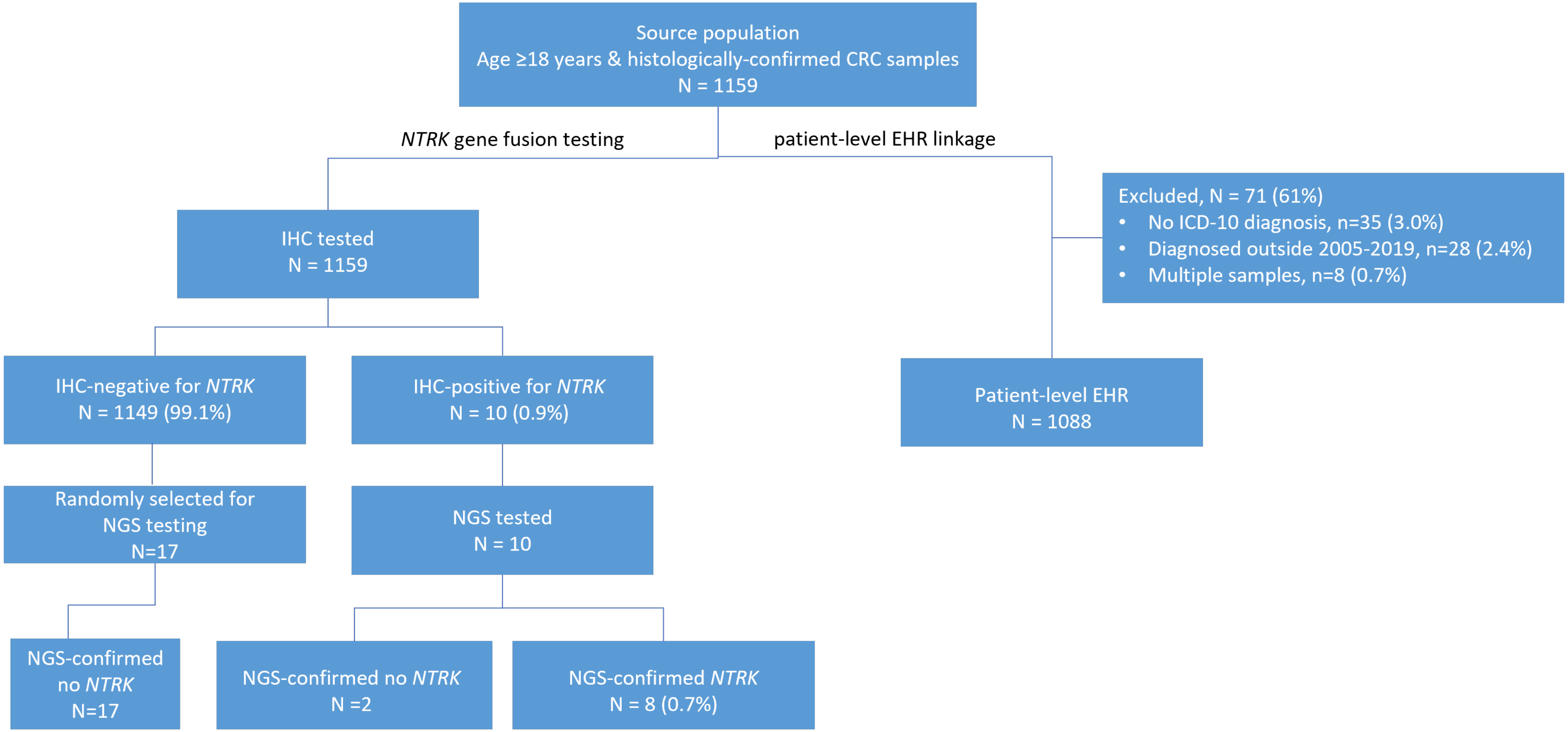

Supplementary Figure 1. Flowchart of CRC patient samples and *NTRK* gene fusion testing.
CRC, colorectal cancer; ICD, International Classification of Diseases; IHC, immunohistochemistry; NGS, next generation sequencing; *NTRK*, neurotrophic tyrosine receptor kinase

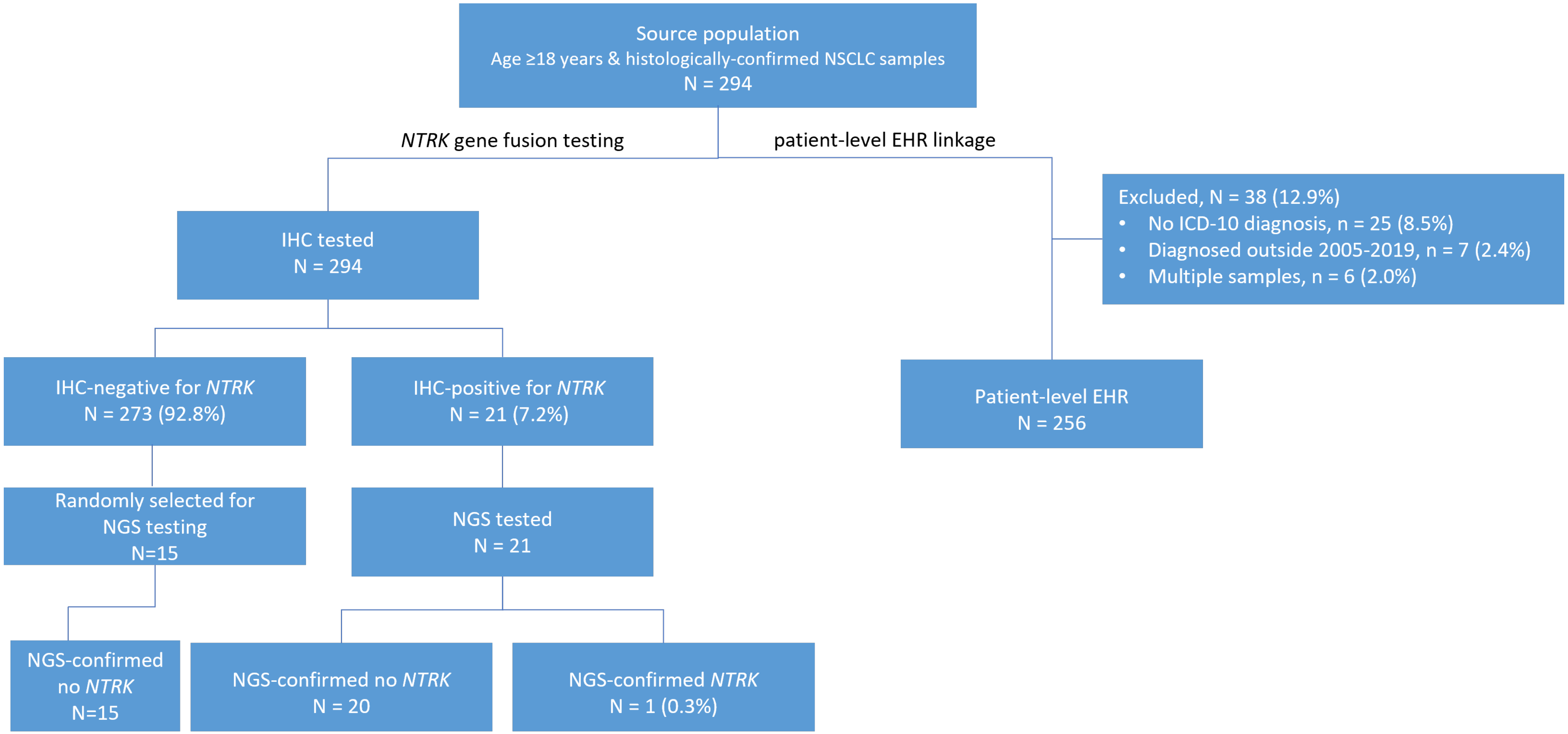

Supplementary Figure 2. Flowchart of NSCLC patient samples and *NTRK* gene fusion testing.
ICD, International Classification of Diseases; IHC, immunohistochemistry; NGS, next generation sequencing; NSCLC, non-small cell lung cancer; *NTRK*, neurotrophic tyrosine receptor kinase

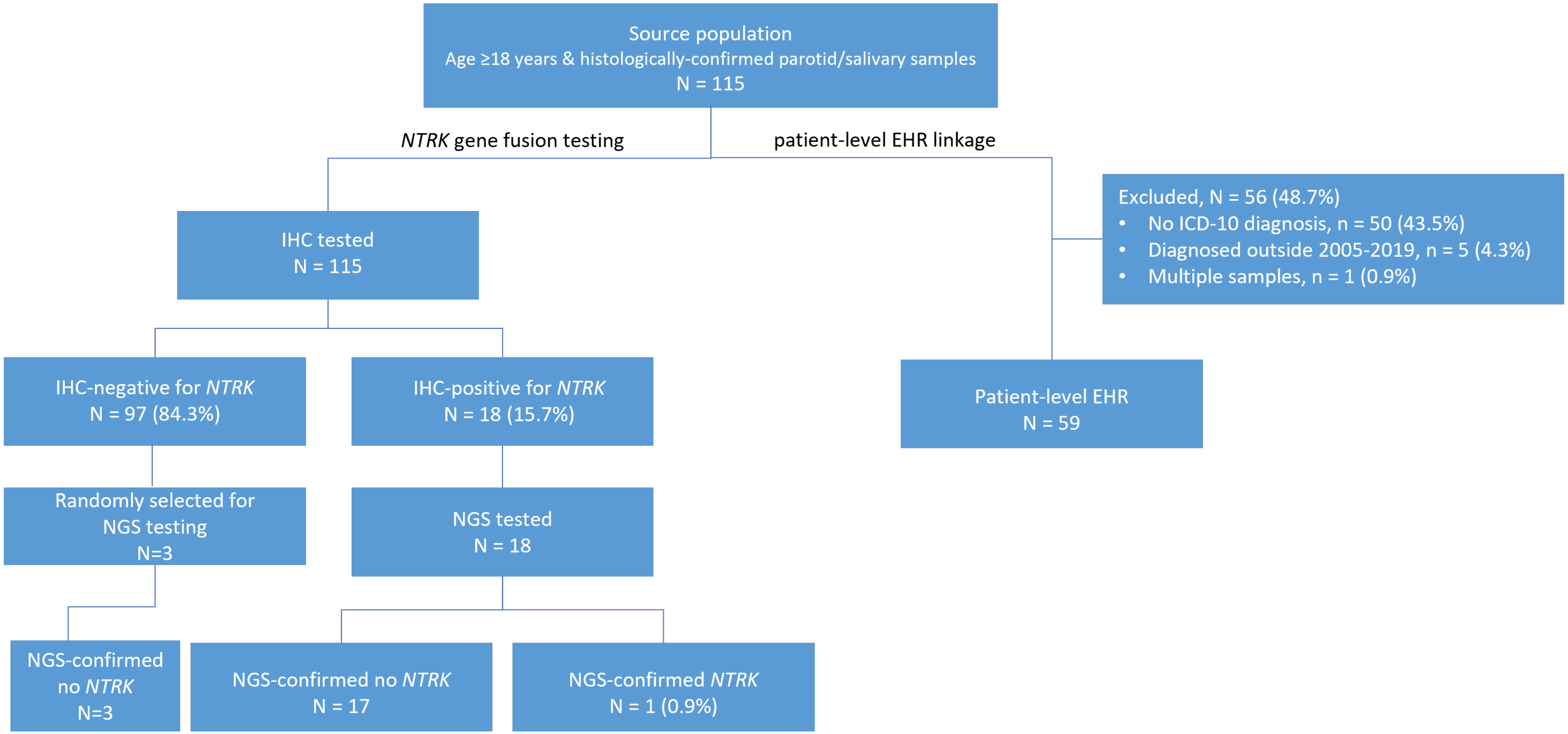

Supplementary Figure 3. Flowchart of salivary cancer patient samples and *NTRK* gene fusion testing.
ICD, International Classification of Diseases; IHC, immunohistochemistry; NGS, next generation sequencing; *NTRK*, neurotrophic tyrosine receptor kinase

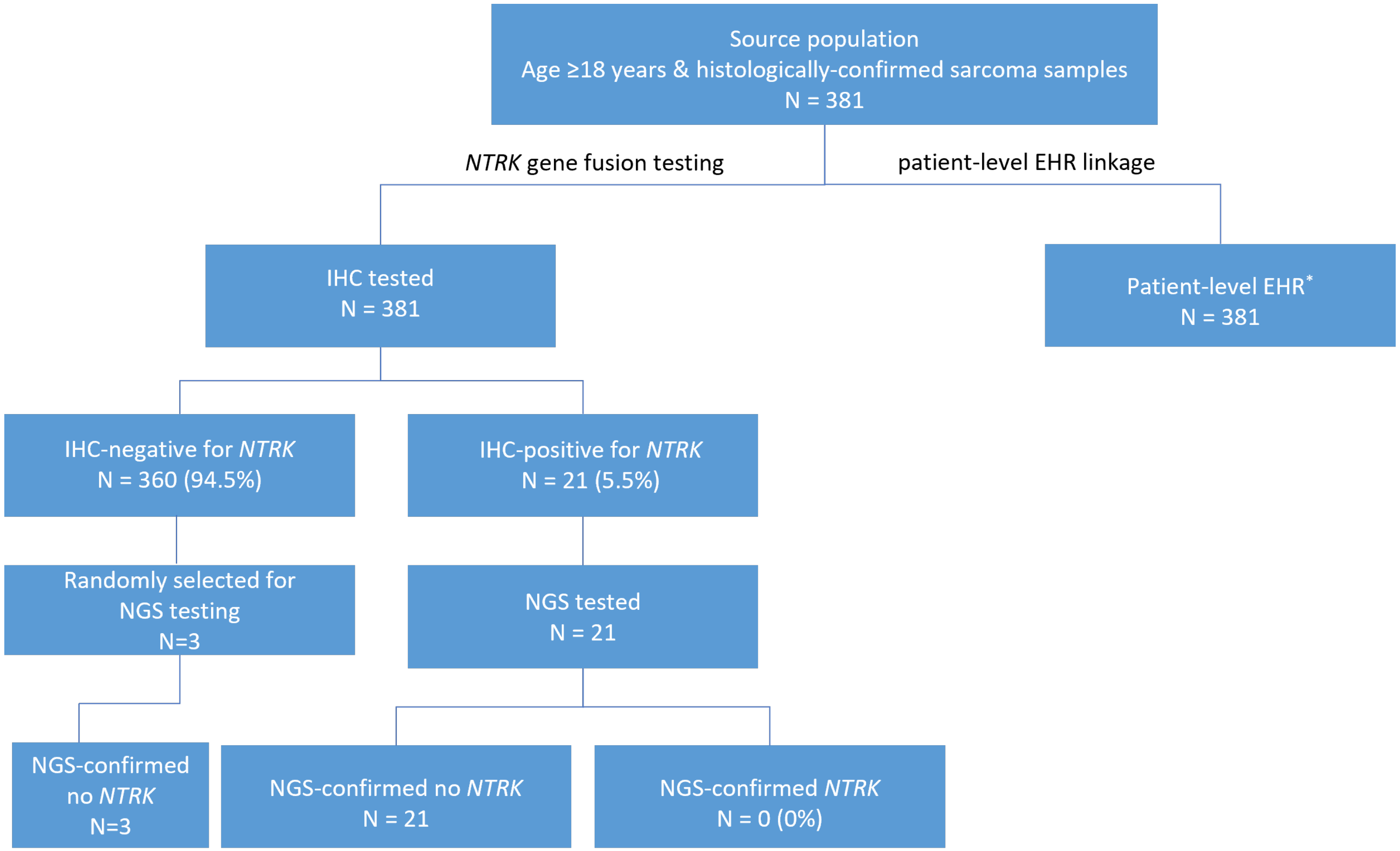

Supplementary Figure 4. Flowchart of sarcoma patient samples and *NTRK* gene fusion testing.
*As no patients were, however, found to be positive for *NTRK* gene fusion, the clinic-genomic part of the study (using EHRs) was not carried out.
ICD, International Classification of Diseases; IHC, immunohistochemistry; NGS, next generation sequencing; *NTRK*, neurotrophic tyrosine receptor kinase

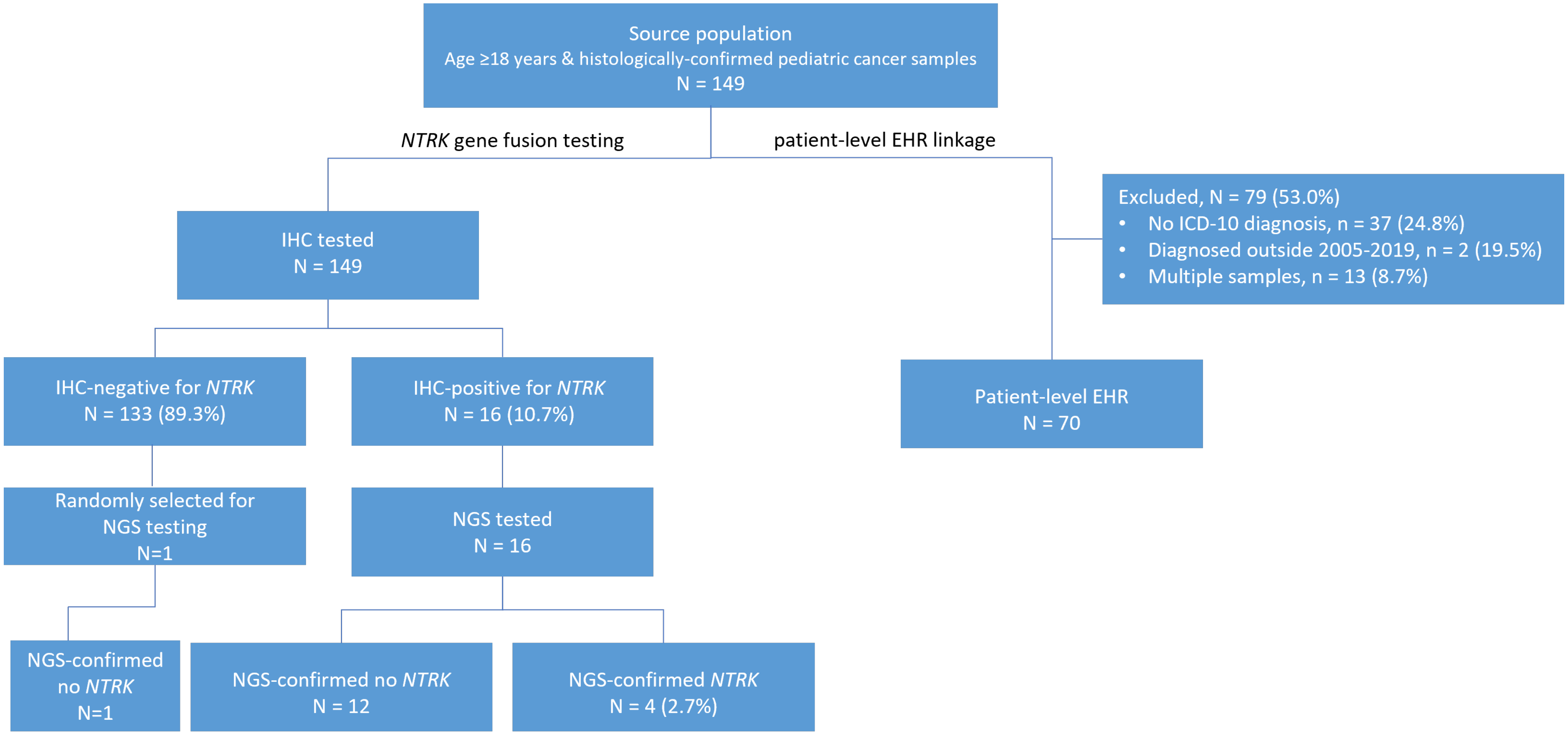

Supplementary Figure 5. Flowchart of pediatric cancer patient samples and *NTRK* gene fusion testing.
ICD, International Classification of Diseases; IHC, immunohistochemistry; NGS, next generation sequencing; *NTRK*, neurotrophic tyrosine receptor kinase

Supplementary Table. Technologies for fusion detection used in the referenced studies.

Reference	Adult CRC	Adult NSCLC	Adult salivary	Adult sarcoma	Any paediatric	Technology for fusion detection	Remarks
Our present study	0.7% (8/1,151)	0.3% (1/288)	0.9% (1/114)	0% (0/379)	3.1% (4/127)	TST170 RNA hybrid capture NGS	N/A
2 (Westphalen *et al*, 2021)	0.22% (77/34,697)	0.24% (136/56,615)	2.43% (35/1,440)	1.27% (79/6,216)	1.34% (59/4,388)	FoundationOne, One CDx, and Heme V4 assays using DNA (and RNA) based adaptor ligation and hybridization capture NGS; unclear which technique for which samples	database study
3 (O'Haire *et al*, 2023)	0.22% (65/29,578)	0.19% (115/60,272)	2.50% (24/962)	0.68% (13/1,915)	2,85% (241/8,470)	Most studies (62%) identified NTRK fusions through RNA and DNA targeted panels; see suppl. info of the ref. for further details	Adult incl. mixed age cohorts; adult are prevalence point estimates derived from a systematic review of 160 original articles
5 (Solomon *et al*, 2020)	0.31% (9/2,929)	0.23% (9/3,993) lung adenocarcinoma only	5.08 (13/256)	0.68% (13/1,915)	N/A	MSK-IMPACT DNA hybridization capture NGS and MSK-Fusion RNA anchored multiplex NGS; unclear which technique for which samples	age of patients not documented, might be mixed age cohorts

Reference	Adult CRC	Adult NSCLC	Adult salivary	Adult sarcoma	Any paediatric	Technology for fusion detection	Remarks
16 (Gatalica *et al*, 2019)	0.16% (2/1,272)	0.10% (4/4,073)	N/A	0.42% (2/478)	N/A	Archer Dx FusionPlex RNA NGS panel	N/A
17 (Bridgewater *et al*, 2022)	0.33% (9/2,693)	0.06% (1/1560)	N/A	0.60% (7/1,174)	2.38% (4/168)	whole genome sequencing using tumor vs germline DNA	tumor type according to Genomics England classification
18 (Okamura *et al*, 2018)	0.97% (3/310) Colon adenocarcinoma only	0.18% (1/541) lung adenocarcinoma only	N/A	0.76% (2/263)	0.34% (12/3,501)	adult TCGA - paired-end RNA sequencing; pediatric RNA sequencing	N/A
20 (Zhao *et al*, 2021)	N/A	N/A	N/A	N/A	2.22% (27/1217)	Custom CHOP Cancer Fusion Panel to identify known fusion genes and potential novel fusion genes using anchored multiplex PCR (ArcherDX) typically on RNA	N/A
